# CUL5 is required for thalidomide-dependent inhibition of cellular proliferation

**DOI:** 10.1371/journal.pone.0196760

**Published:** 2018-05-10

**Authors:** Bryan Kunkler, Daniel Salamango, Zachary J. DeBruine, Caitlin Ploch, Shirley Dean, David Grossens, Michael P. Hledin, Gabriel A. Marquez, Julie Madden, Abigayle Schnell, Michael Short, Maria A. Burnatowska-Hledin

**Affiliations:** 1 Department of Chemistry, Paul A. Schaap Science Center, Hope College, Holland, MI, United States of America; 2 Department of Biology, Paul A. Schaap Science Center, Hope College, Holland, MI, United States of America; Thomas Jefferson University, UNITED STATES

## Abstract

Angiogenesis is essential for cancer metastasis, thus the discovery and characterization of molecules that inhibit this process is important. Thalidomide is a teratogenic drug which is known to inhibit angiogenesis and effectively inhibit cancer metastasis, yet the specific cellular targets for its effect are not well known. We discovered that CUL5 (previously identified as VACM-1), a scaffold protein in E3 ligase complexes, is involved in thalidomide-dependent inhibition of endothelial cell growth. Our results show that in human endothelial cells (HUVEC), thalidomide-dependent decrease in cell growth was associated with decreased nuclear localization of CUL5. In HUVEC transfected with anti-VACM-1 siRNA, thalidomide failed to decrease cell growth. Previously it was established that the antiproliferative effect of CUL5 is inhibited in rat endothelial cells (RAMEC) transfected with mutated CUL5 which is constitutively modified by NEDD8, a ubiquitin-like protein. In this study, the antiproliferative response to thalidomide was compromised in RAMEC expressing mutated CUL5. These results suggest that CUL5 protein is involved in the thalidomide-dependent regulation of cellular proliferation in vitro. Consequently, CUL5 may be an important part of the mechanism for thalidomide-dependent inhibition of cellular proliferation, as well as a novel biomarker for predicting a response to thalidomide for the treatment of disorders such as multiple myeloma and HIV infection.

## Introduction

Cullin-5 (CUL5), first cloned as Vasopressin-Activated Calcium-Mobilizing (VACM-1) protein [1,2] is the least conserved member of the cullin family of proteins [3,4] used in the formation of the active cullin-RING ubiquitin E3 ligase complexes (CRLs) [5,6]. The numerous E3 ligases that have been identified determine the specificity and diversity of the ubiquitin-proteasome system responsible for the non-lysosomal degradation of proteins. To activate CRLs, cullins are covalently modified at the carboxy-terminus with an ubiquitin-like protein, NEDD8 (Neural precursor cell-expressed developmentally down-regulated) through a process known as neddylation [7–9].

CUL5 is distinct from other cullins both in sequence and tissue distribution [3, 10], but like other cullins it can form E3 ligase complexes involved in ubiquitination and subsequent degradation of specific proteins [6]. The best characterized substrate for the CUL5-specific CRL activity is the APOBEC3G protein essential in the prevention of HIV infectivity [11–13]. To induce destruction of APOBEC3G, the CUL5-CRL forms complexes with the adaptor proteins elongins B/C and with the substrate receptor protein Vif. Whereas elongins B/C are the recognized adaptor proteins for the CUL5-based CRLs, several CUL5-specific substrate recognition receptor-proteins have been identified and include viral proteins Vif and E4orf6, HSP90, Dab1, ASB2 and XRAB40 [14–18]. Unfortunately, the mechanism by which substrate recognition proteins recruit specific targets for CUL5-dependent CRL activity, *in vivo*, have not yet been explored.

Human CUL5 localizes at chromosome 11 to the region at 11q22-23 which is frequently deleted in breast cancer [2]. A significant decrease in CUL5 expression is observed in the majority of breast cancers [19,20], while inhibition of CUL5 expression using microRNA-19a and -19b induced cervical carcinoma cell proliferation and invasion [21]. The inhibition of cell growth by CUL5 depends on controlling signaling pathways that may be cell type-specific. For example, overexpression of CUL5 cDNA in a breast cancer-derived cell line, T47D, inhibited nuclear localization of estrogen receptor ERα and inhibited estrogen-dependent cell growth [22]. In rat adrenal medullary endothelial cells (RAMEC), overexpression of CUL5 affected the expression of maspin [23] and in COS-1 cells it induced p53 and decreased aquaporin 1 (AQP1) expression, proteins implicated in the control of cancer [24,25]. In all cell types tested, overexpression of CUL5 cDNA significantly reduced concentrations of phosphorylated MAPK (mitogen activated kinase) but did not affect total MAPK concentrations [22–25]. More recently, an activated Src protein has been identified as a new target for CUL5 dependent degradation [26] while silencing CUL5 reduced cellular sensitivity to HSP90 inhibitors [27].

CUL5 is expressed in many tissues, but it is highest in the heart and skeletal muscle [10], tissues where highest expression of NEDD8 has also been shown [7]. Our immunocytochemistry data shows that in most tissues examined, expression of CUL5 protein localizes specifically to vascular structures and to the renal collecting tubule cells [23, 25]. The endothelium-specific role for cullins is supported by a report that targeted disruption of the CUL7 gene leads to abnormal vascular morphogenesis [28], and CUL5 expression is required for the degradation of HIF and ERBB2 proteins implicated in the induction of pro-angiogenic signaling pathways [14]. Interestingly, in a rat model of experimentally-induced angiogenesis *in vivo* we observed that CUL5 expression was very high in non-proliferating blood vessels and was largely absent in blood vessels during exercise-induced angiogenesis [23]. Unfortunately, few studies have addressed the pathophysiological significance of tissue-specific expression of CUL5 *in vivo* [29].

In our search for compounds that regulate expression of CUL5, we have focused on thalidomide, a drug that inhibits cellular growth and angiogenesis [30]. Thalidomide, a derivative of glutamic acid, is a drug first used in the late 1950s to ease morning sickness, but was banned after it was discovered to be a teratogen [30, 31]. The cellular mechanism of thalidomide-induced cell death encompasses a broad range of signaling pathways that may be different in various species and cell types [32–36]. For example, thalidomide inhibits angiogenesis but may induce vessel maturation rather than inhibiting sprouting capillaries [30,37]. Thalidomide and its derivatives, lenalidomide and palidomide, have been used effectively in treatments of several types of cancer, HIV/AIDS, Crohn’s disease, congestive heart disease, and many others [7, 32–39]. More recently, CRBN (cereblon), a candidate gene for mild mental retardation protein, has been identified as a novel target for thalidomide teratogenicity [40]. Binding of thalidomide to CRBN was shown to inhibit CUL4A-dependent E3 ligase activity, and thalidomide promoted CRBN-dependent degradation of specific transcription factors that play a critical role in B cell malignancies including myeloma [41,42]. It has also been reported that that majority of myeloma cell lines from patients resistant to thalidomide and other immunomodulatory drugs have very low levels of CRBN [43,44]. Whether other cullins can be a target for thalidomide-specific activity has not been explored.

In the studies reported here, we show that in normal endothelial cells (HUVEC and RAMEC), the antiproliferative effect of thalidomide was dependent on presence ofCUL5 and was associated with decreased nuclear localization of CUL5 protein. The antiproliferative response to thalidomide was also compromised in RAMEC transfected with mutated CUL5 cDNA shown previously to be constitutively neddylated and to reverse the antiproliferative effect of wild-type CUL5 cDNA overexpression [23–24]. Our immunocytochemistry results also showed that in HUVEC treated with thalidomide, nuclear NEDD8 signal was attenuated, whereas its cytosolic signal was increased. Together, these results confirm the regulatory role of CUL5 in the angiogenic pathway and identify it as a novel target in thalidomide-directed therapy. Importantly, the expression of CUL5 could be used as a biomarker for predicting a specific response to thalidomide and possibly other anti-angiogenic drugs used clinically.

## Materials and methods

### Materials

HUVEC (several lots) were purchased from Invitrogen Co. (Grand Island, NY), and RAMEC were a gift from Dr. Lelkes [45]. Thalidomide (±) (T144], ECGS (endothelial cell growth supplement), PMA and anti-NEDD8 specific monoclonal antibody were purchased from Sigma-Aldrich (St. Louis, MO). The polyclonal anti-NEDD8 Ab was purchased from Abcam (Cambridge, MA). AlamarBlue® reagent was purchased from Biosource (Invitrogen Co., Grand Island, NY). The siRNA Starter Kit (*Silencer*®) was purchased from Ambion® (cat # AM16708A). All tissue culture media and reagents were purchased from Invitrogen (Grand Island, NY). ^S730A^CUL5 (previously called ^S730A^VACM-1) cDNA used to transfect RAMEC was sublconed into the pBK-CMV vector (Stratagene Co., La Jolla, CA) as described previously [23,24].

### Tissue culture

HUVEC cells were grown in F-12K liquid media supplemented with 20% FBS, ECGS (30 μg/ml), 1% Pen/Strep, and heparin (0.5mg/ml), or medium 200 supplemented with as suggested by the supplier (Invitrogen Co., Grand Island, NY). RAMEC cells were grown in DMEM-low glucose media supplemented with 2% FBS and 8% horse serum. Cells were plated at a density of 4-6x10^5^ cells per 100 mm plate and maintained at 37°C under water-saturated 5% CO_2_ atmosphere as previously described [24]. Thalidomide and PMA (10 nM) (both from Sigma-Aldrich) were diluted in DMSO and control cells were treated with equal amounts of DMSO.

### Cell growth assay

Cell growth was monitored using either DAPI staining (Vector Labs) or the alamarBlue^®^ (Invitrogen Co.) assay as described in the manufacturer’s instructions. For the 96-well plate growth assay, HUVEC were plated at a range of 1x10^3^ to 5x10^3^ cells/mL. After treatments, alamarBlue® reagent was added at 10% the total well volume. Fluorescence readings at 560 and 590 nm were taken at time intervals of 2, 4, 8, 12, 18, 24, and 48 hours post treatment.

### Wound healing growth assay

Cells were plated on 6-well tissue culture plates at 5x10^4^ cells/mL. After cells reached confluency, the cell layer was scratched using a 200 μl pipette tip. Cell cultures were photographed at time 0 and at specified time points after the appropriate treatments. *Image-pro express®* was used to measure wound distance in the cell monolayer. To correct for uneven wound between groups, all growth data are expressed as a percent (%) change from time 0.

### Immunocytochemistry

Affinity purified rabbit polyclonal antibody directed against the C-terminus (Ab-B) of CUL5 protein [1] was used to stain cells by indirect immunofluorescence. Cells grown on coverslips were fixed in 3% paraformaldehyde (in 1xPBS, pH 7.4) for 20 minutes, washed in PBS, permeabilized with 0.5% Tween-20 solution for 20 minutes, washed with PBS/2% BSA, and incubated for two hours with a 1:20 dilution of Ab-B. The specificity of our antibodies has been assessed previously using Ab-B preabsorbed with 10 μM peptide B identical in sequence to the carboxyl terminus sequence of CUL5. Anti-NEDD8 antibody (Sigma) was diluted 1:200. All antibodies were diluted in PBS containing 0.1% BSA (PBS/BSA). The primary antibodies were detected by incubating cells in the presence of 1:40 dilution of either FITC-conjugated goat anti-rabbit IgG or Texas Red conjugated anti-rabbit Ab (Vector Laboratories Inc, Burlingame, CA) in 1X PBS/2% BSA for 1 hr. The slides were washed with 1x PBS with 0.2% BSA, mounted with Vectashield^®^ mounting medium (Vector Laboratories Inc, Burlingame, CA) and viewed by epifluorescence microscopy (Eclipse E600, Nikon) equipped with Spot camera (Diagnostic Instruments, Sterling Heights, MI). The nuclear staining was achieved by DAPI found in the Vectashield^®^ mounting medium.

### siRNA transfections

Transfections were performed with anti-VACM-1 siRNAs targeting different regions of the CUL5 mRNA sequence according to the protocol in the *Silencer*® siRNA Starter Kit purchased from Ambion® (cat # AM16708A) as described previously [46]. Briefly, the pre-designed *Silencer*® siRNA kit contained three specific antisense oligonucleotides: siRNA#1 5’-AGAUUCCUGGCGUAAAAGCtt-3’ (ID 192207), siRNA#2 5’-CCACGUAUCAA GCAUGAGCtt-3’ (ID 192208), and siRNA#3 5’-UAGCAUCAUUAACAACUGCtt-3’ (ID 192209). For the negative control, cells were transfected with siRNAs that did not target any gene sequences. The positive control used was an anti-GAPDH siRNA provided in the starter kit.

### Cell growth on Matrigel^®^

The Matrigel^®^ (BD Biosciences, Bedford MA) was warmed to room temperature, 100 μl was placed onto coverslips in a 6-well plate. Matrigel^®^ support medium was then placed in an incubator at 37°C for 10 to 15 minutes in order to polymerize. HUVEC were plated at 4x10^4^ cells/mL and incubated for 30 minutes. After incubation, 1.4 mL of fresh media was added to each well and incubated overnight. A transfection with anti- CUL5-specific siRNA was performed before cells were plated on Matrigel^®^ support according to the Ambion^®^ transfection protocol and cells were treated with thalidomide at 24 hours after transfection (n = 3).

### Western blot analysis

Total cell lysates were prepared as previously described [24]. Briefly, cells were grown to at least 70% confluency, washed with ice-cold PBS, and resuspended in 200 μl of buffer (50mM Tris [pH 7.4], 0.1% Triton X-100, 150 mM NaCl, 1 M EDTA, 50 mM NaF with 1 μg/ml apoprotein, 100μM Prefabloc SC, and 10 mM PMSF. Protein concentrations were determined using the Bradford method (BioRad Co., Richmond, CA). Samples were heated at 75°C for 5 minutes and subjected to SDS polyacrylamide gel electrophoresis (PAGE) using a 4–12% bis-tris running gel (Invitrogen Inc). The separated proteins were transferred to a nitrocellulose membrane (Osmotics Co. Trevose, PA) at 20 mV for 2 hours. Nonspecific sites were blocked using PBS with 5% nonfat dry milk for 45 minutes. The membranes were next incubated for 2 hours in buffer with 1:200 dilution of affinity purified polyclonal antibodies directed against the N-terminus (Ab-A) of CUL5 (VACM-1) protein [1]. After incubation with the primary antibody, the membrane was washed for twenty minutes in buffer used to dilute antibody and exposed to horseradish peroxidase conjugated secondary antibodies diluted 1:2000 (Cell Signaling, Beverly, MA). The membranes were exposed to luminal detection reagents (Cell Signaling, Beverly, MA) for 1 minute or longer, and exposed to the x-ray film (Amhersham). To ascertain equal protein loading on the gel, blots were stripped and re-probed with a GAPDH specific antibody (1:5000) developed in mouse and the signal for VACM-1/CUL5 was corrected for GAPDH levels.

### Statistical analysis

All immunoblots and Western blots were scanned and the signal was quantitated using the NIH Imaging Analysis Program (http://rsb.info.nih.gov/ij/index.html). Data are expressed as means ± one standard error (SE) of the mean. Student’s t-test was used for data analysis. Significance was set at *p* < 0.05.

## Results

### Expression of CUL5 in HUVEC cells is induced by thalidomide

**Fig 1:**
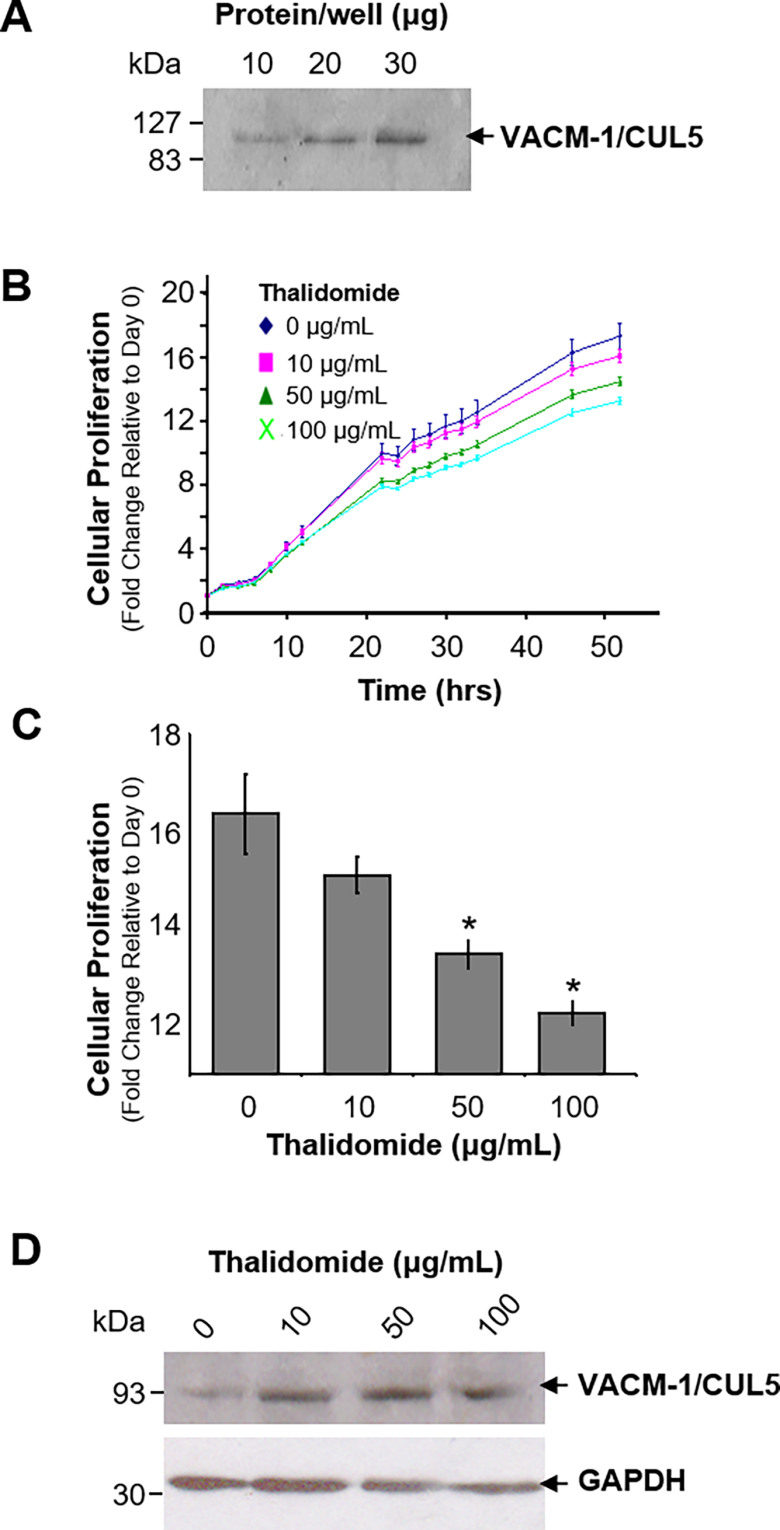
Effect of thalidomide on cellular growth and CUL5 protein expression in HUVEC. **A.** Western blot analysis of CUL5 protein expression in HUVEC. Increasing concentrations of protein were loaded on the SDS-PAGE and after transfer to nitrocellulose blots were probed with anti CUL5-specific antibody. **B.** Dose and time-dependent effects of thalidomide on growth in HUVEC (*n* = 3, in triplicate). **C.** The proliferation data at 48 hours shown in B were re-plotted as a bar graph (*n* = 3, *, *p* < 0.05). **D.** Western blot analysis of cell lysates from HUVEC treated increasing concentrations of thalidomide. Upper panel: CUL5 protein expression in thalidomide treated cells. Lower panel: To ascertain equal protein loading, blots were re-probed for GAPDH.

**Fig 2:**
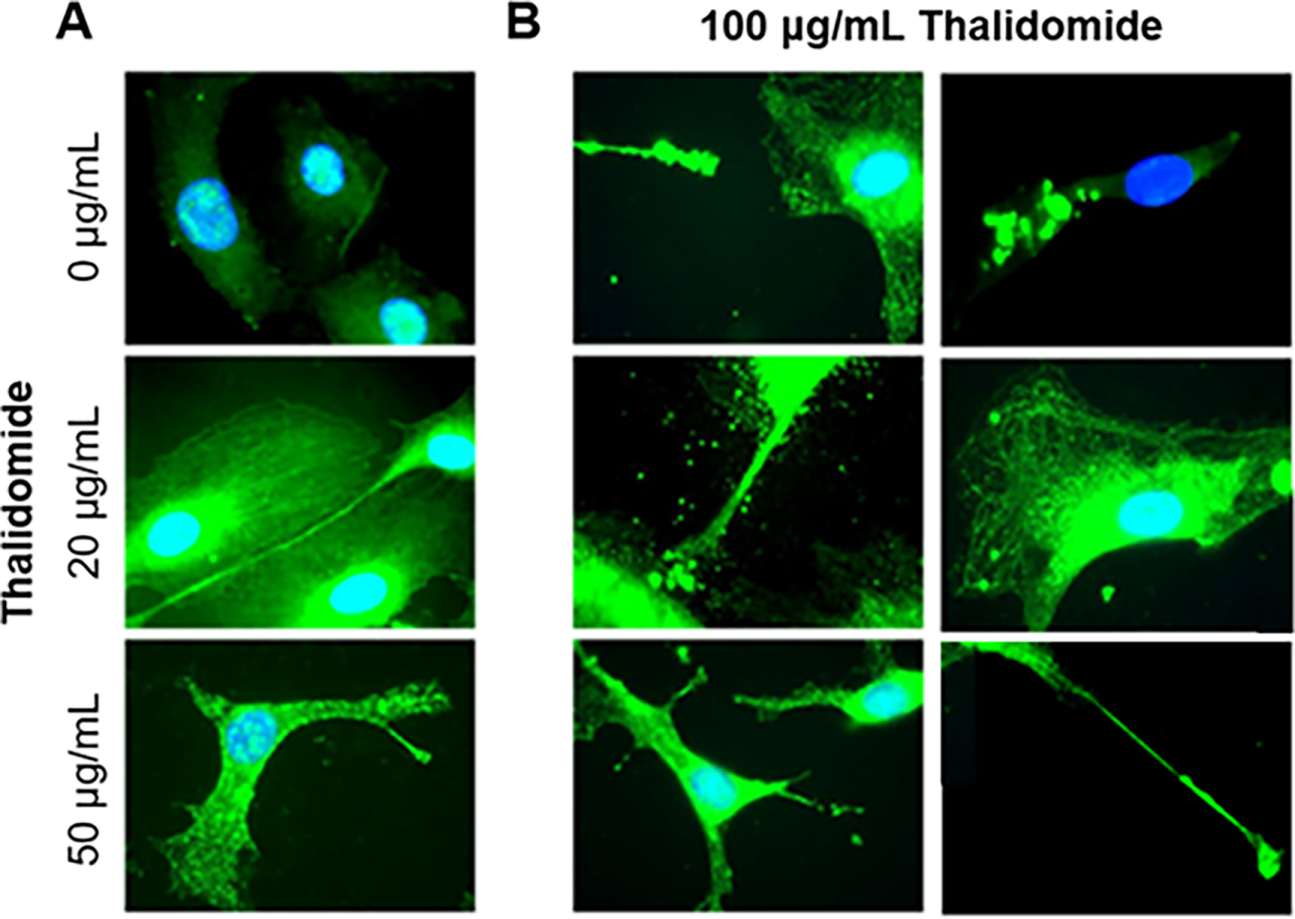
CUL5 protein localization in HUVEC treated with increasing doses of thalidomide. **A.** The control and thalidomide-treated HUVEC (20 and 50 μg/ml). **B.** Examples of HUVEC treated with 100 μg/ml thalidomide. Cells were immunostained using anti- CUL5 specific antibody as described in the Methods. Magnification is 100X.

We examined the possibility that CUL5 may be involved in the antiproliferative and anti-angiogenic effects of thalidomide in human endothelial cells *in vitro*. First, the expression of CUL5 protein in HUVEC was confirmed by Western blot (Fig 1A) as described previously [1]. Next, dose and time dependent effects of thalidomide on HUVEC growth were measured over 50 hours using a colorimetric growth assay. As expected, treatment with thalidomide inhibited cell growth in a dose dependent manner (Fig 1B and 1C). Western blot analysis suggested that CUL-5 concentration was also increased at 24 hours post thalidomide treatment (Fig 1D) but the increase was not significant. We also observed that thalidomide induced changes in cell morphology and the formation of putative lamellipodia [37] were associated with redistribution of CUL5 protein from the nucleus to the cytosol and possibly to the cell membrane (Fig 2). These effects of thalidomide were dose-dependent: vesicular trafficking of CUL5 towards the cell membrane was observed at 20 μg/mL of thalidomide, while at 50 and 100 μg/ml μg/ml of thalidomide, the cells became elongated with extensive protrusions toward neighboring cells (Fig 2A and 2B). These results provide the first evidence that in addition to CUL4 CRLs, thalidomide may also control activation of CUL5-dependent CRLs, at least in endothelial tissues.

### Depletion of CUL5 in HUVEC using siRNAs reduces response to thalidomide

**Fig 3:**
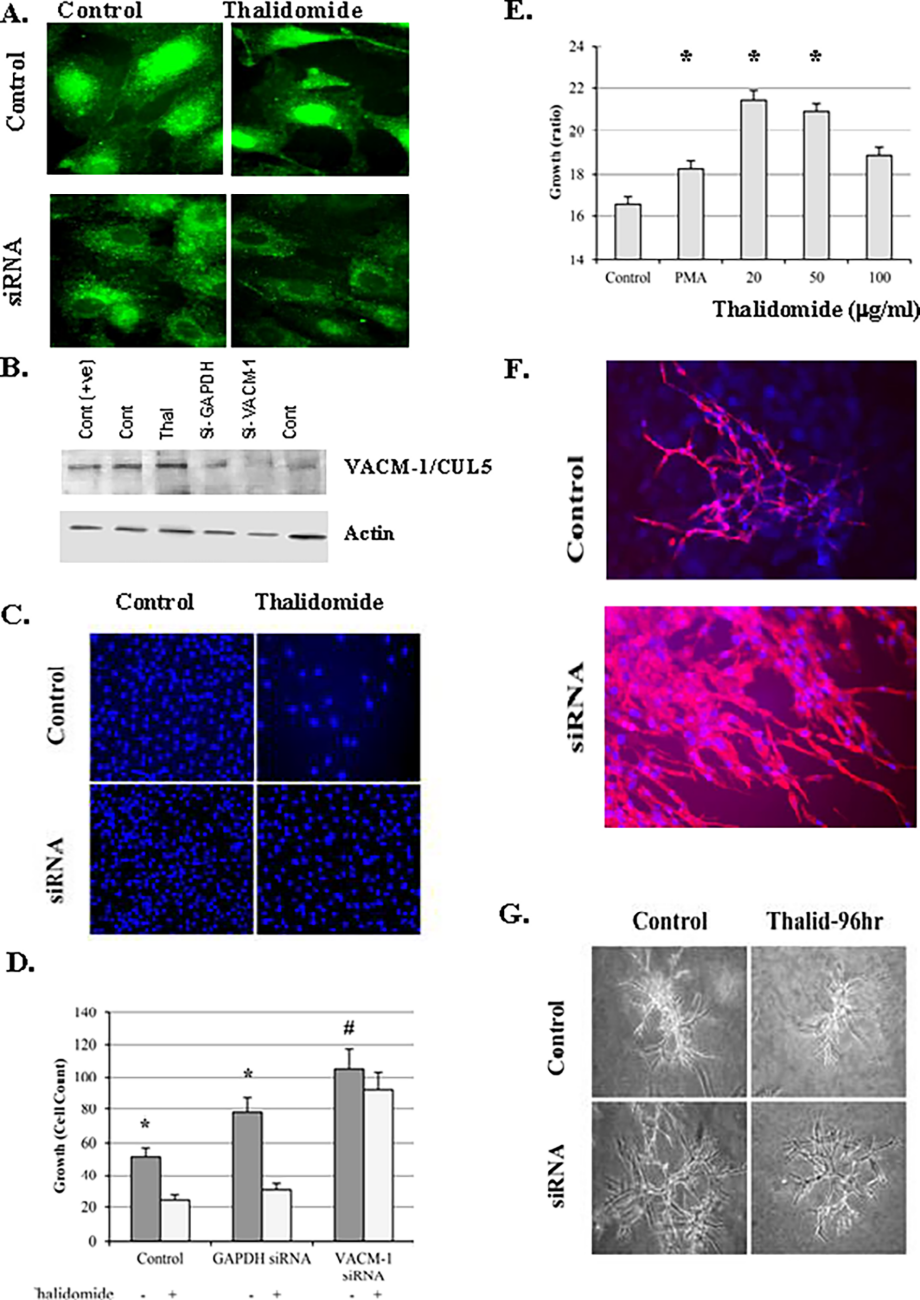
siRNA-mediated depletion of CUL5 in HUVEC prevents thalidomide-dependent decrease in cellular proliferation. **A.** A representative immunocytochemistry results showing CUL5 distribution in control and thalidomide (50 μg/ml) treated HUVEC that were sham transfected or transfected with anti-VACM-1 specific siRNA. Magnification is 100X. **B.** Western blot analysis of cell lysates from control (Cont), thalidomide (thal) and siRNA-GAPDH and siCUL5 transfected HUVEC probed with anti-VACM-1/CUL5 Ab A. A sample of lysate from ^S730A^VACM-1/CUL5 transfected RAMEC was used as a positive control (Cont(+ve)). Blots were subsequently re-probed using anti-actin antibody. **C.** DAPI nuclear staining of control-siRNA and anti-CUL5 si-RNA transfected HUVEC treated with thalidomide (50 μg/ml) for 24 hours. Magnification is 10X. **D.** Cellular count using DAPI-stain signal for the experiment shown in C (*, p<0.05 for cell growth in thalidomide treated vs control cells. #, p<0.05 when cell counts in control-siRNA and anti-CUL5 si-RNA transfected cells were compared). **E.** Effects of thalidomide on siRNA-transfected HUVEC growth using alamarBlue^®^ assay. PMA (10^−7^ M) was used as a control (n = 3, *, p<0.05 when compared to control). **F.** Control and siRNA-transfected HUVEC grown on Matrigel^®^ coated plates. Magnification is 20X. **G.** A representative experiment showing effects of thalidomide in control and siRNA-transfected HUVEC grown on Matrigel^®^ coated plates. Magnification is 10X.

We next hypothesized that if the antiproliferative effect of thalidomide was dependent on CUL5, HUVEC treated with anti-CUL5-specific siRNAs would be less responsive to thalidomide. We thus repeated the immunostaining and proliferation experiments in HUVEC transfected with siRNA designed against CUL5 that we used previously [46]. All three available siRNAs were used in one experiment, and subsequently, all experiments were performed using siRNA#1 alone or a combination of siRNAs 1 and 2. Our immunocytochemistry results confirmed a decrease in CUL5 signal in the siRNA transfected HUVEC when compared to controls (Fig 3A). Furthermore, in the siRNA transfected cells, thalidomide treatment (50 μg/ml) did not appear to change the shape of the cell observed in the control HUVEC (Fig 3A). The decrease in CUL5 signal in siRNA transfected HUVEC was also confirmed by the Western blot analyses (Fig 3B). The effects of thalidomide on cellular proliferation in control, GAPDH siRNA and anti-VACM-1/CUL5 siRNA transfected cells were examined next. Both, DAPI nuclear staining count (Fig 3C and 3D) and alamarBlue^®^ proliferation assay (Fig 3E) were used to monitor cell growth. Our results suggest that treatment with anti-CUL5-specific siRNA for 24 or 48 hours caused an increase in cell growth when compared to the anti-GAPDH siRNA or the sham transfected group (Fig 3C and 3D). Additionally, in HUVEC transfected with anti-CUL5 siRNA where an increase in cell proliferation was observed, thalidomide treatment did not inhibit cell growth (Fig 3D and 3E). The involvement of CUL5 in thalidomide-dependent control of cellular growth was further confirmed when cells were grown on a Matrigel^®^ support for 4–5 days. The knockdown of CUL5 transcripts with siRNA enhanced formation of “angiogenic branches” (Fig 3F) and reduced the inhibitory effect of thalidomide on “cord formation” when compared to controls Fig 3G). These results further underscore the importance of CUL5 in the control of endothelium-specific effects of thalidomide.

### The antiproliferative effect of thalidomide is dependent on the neddylation status of CUL5

**Fig 4:**
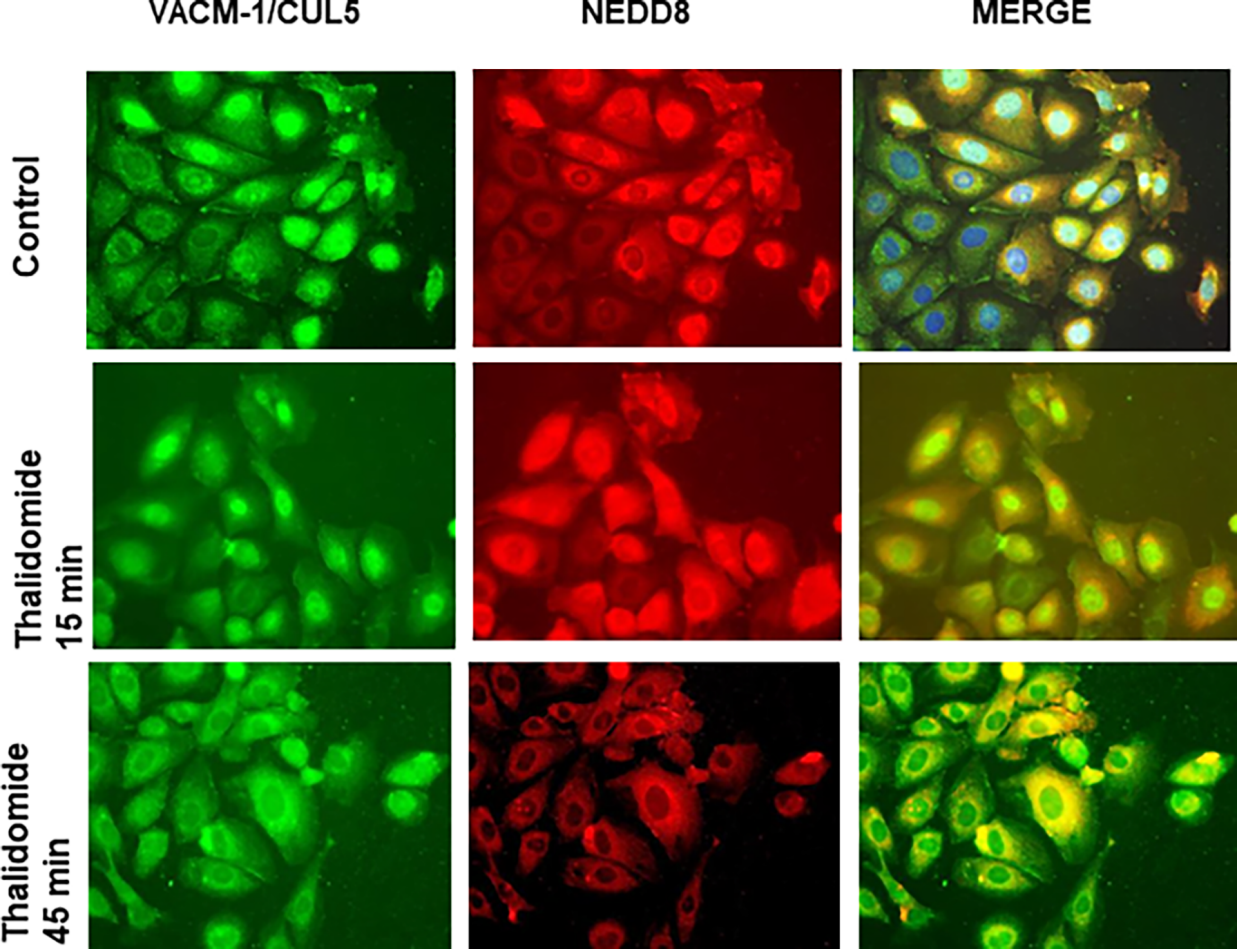
Effect of thalidomide on CUL5 and NEDD8 localization in HUVEC. CUL5 and NEDD8 colocalization in control cells treated with thalidomide (50 μg/mL) for 15 and 45 min, respectively. Immunostaining with anti- CUL5 and anti NEDD8 antibodies and nuclear DAPI staining was performed as described in the *Methods*. Images are of merged pictures from CUL5 staining (FITC-green) and NEDD8 staining (Texas red). Magnification is 40X.

**Fig 5:**
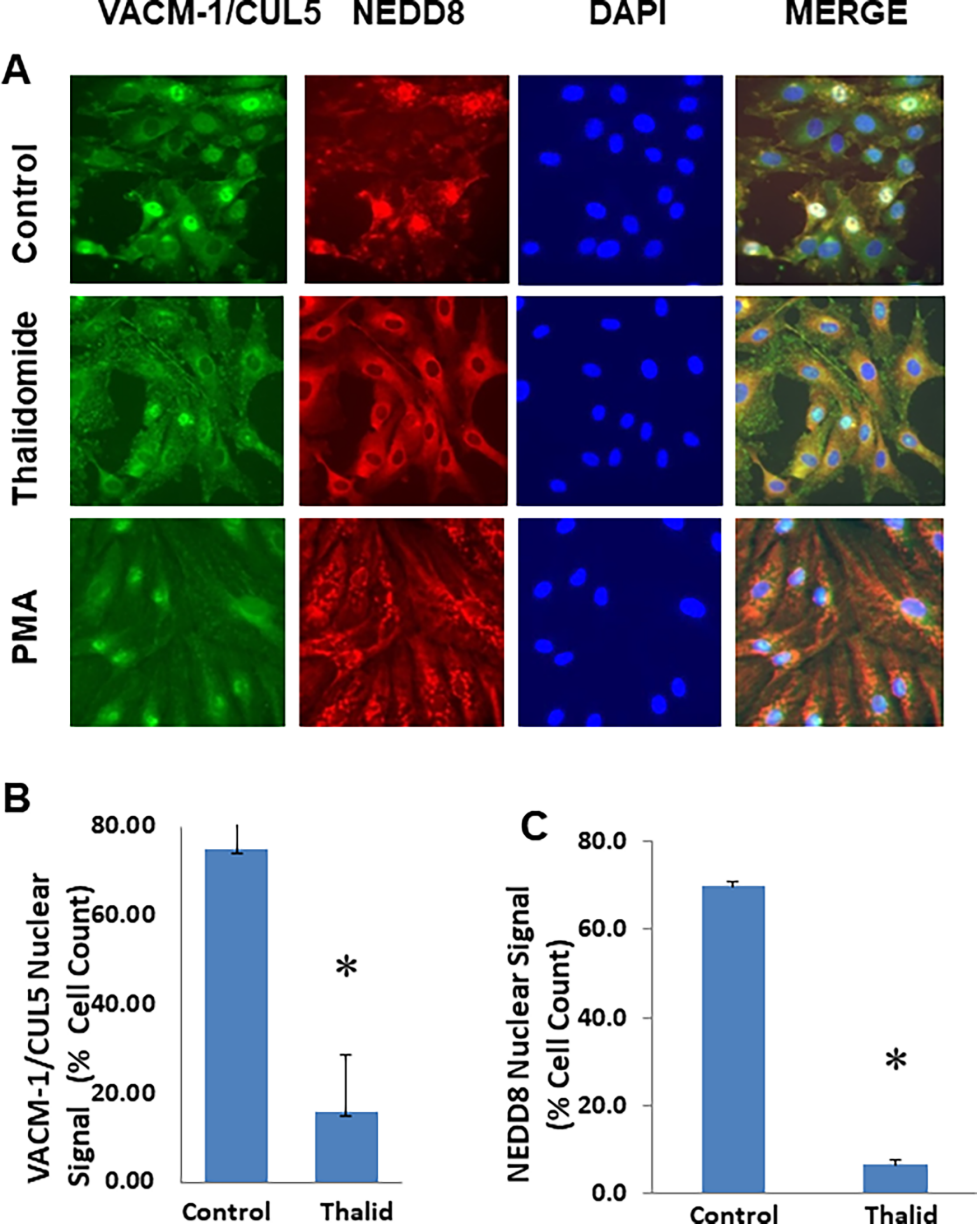
Effect of thalidomide on CUL5 and NEDD8 localization in HUVEC after 24 hrs of treatment. **A.** Expression of CUL5 and NEDD8 in Control, Thalidomide (20 μg/ml) and PMA (10 nM) treated HUVEC for 24 hours. Immunostaining with anti-VACM-1/CUL5 and anti NEDD8 antibodies was performed as described in the Methods. Images are from CUL5 staining (FITC-green) and NEDD8 staining (Texas red) and merged pictures. Magnification is 40X. **B.** Control and thalidomide treated cells expressing nuclear CUL5 and NEDD8 were quantitated (n = 10 and n = 3, respectively; *, p <0.05).

**Fig 6:**
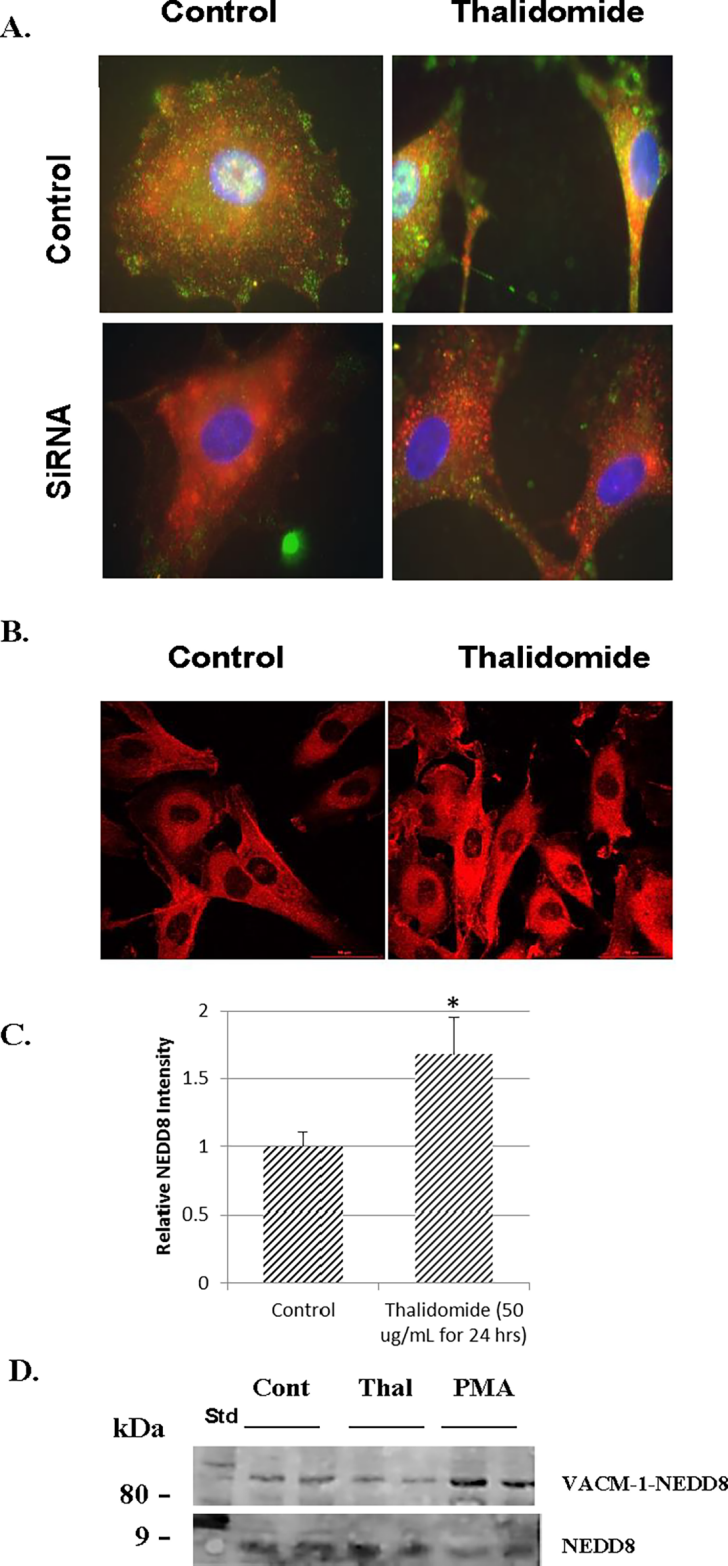
Effect of thalidomide on CUL5 and NEDD8 localization in control and si-transfected HUVEC at 24 hours after treatment. **A.** CUL5 (green) and NEDD8 (red) localization in control and si-CUL5 transfected HUVEC and treated with thalidomide (50 μg/mL) for 24 hrs. Magnification is 100X. **B.** HUVEC treated with thalidomide (50 μg/mL) immunostained with anti NEDD8 Ab. Magnification is 40X. **C.** NEDD8 signal in Control and Thalidomide treated cells shown in B was quantitated (*n* = 3; error bars are S.E.M., *, *p* < 0.05). D. Western blot analysis of neddylated CUL5 (upper band) and free NEDD8 (lower band) in control, thalidomide and PMA-treated HUVEC (24 hours).

Our previous work suggested that the antiproliferative effect of CUL5 is dependent on its posttranslational modification by NEDD8 [24,45]. Recently, a CUL4A-specific ligase has been implicated in thalidomide-dependent anticancer effects [40, 41] but the role of the neddylation in this process has not yet been explored. Thus, we next examined the effect of thalidomide on NEDD8 expression and its co-localization with CUL5 in HUVEC after 15 and 45 min (Fig 4) and 24 hrs (Fig 5) of treatment. In control proliferating cells (“edge of the “wound”), both CUL5 and NEDD8 localized to the nucleus (Fig 4A, top panel). There we no changes in the cellular localization of either protein after 15 min of treatment (Fig 4A, middle panel), and nuclear signal intensities of both, CUL5 and NEDD8, were decreased after 45 min of treatment with thalidomide (Fig 4A, bottom panel). At 24 hrs after treatment, co-immunostaining with anti-CUL5 and anti-NEDD8 antibodies indicated that thalidomide reduced nuclear VACM-1/CUL5 and NEDD8 signal intensity significantly and directed their localization to the cytosol (Fig 5A and 5C). The specificity of the thalidomide effect is further underscored by our observation that PMA (phorbol myristate acetate, or 12-0-tetradecanoyl-phorbol-13-acetate) (10 nM) a tumor-promoting ester treatment attenuated CUL5 but not NEDD8 signal intensity (Fig 5A, bottom panel). Subsequent immunostaining experiments demonstrated that anti-VACM-1/CUL5 siRNA induced decrease in CUL5 signal was not associated with a decrease in NEDD8 signal (Fig 6A). When NEDD8 signal was quantitated, it was significantly higher in thalidomide treated cells was when compared to the controls (Fig 6B and 6C), while Western blot analysis showed a reduced NEDD8-CUL5 signal in HUVEC treated with thalidomide (Fig 6D). Together, these results suggest that thalidomide treatments targets CUL5 modification by NEDD8.

### The expression of a CUL5 dominant negative mutant cDNA prevents antiproliferative effect of thalidomide in RAMEC

**Fig 7:**
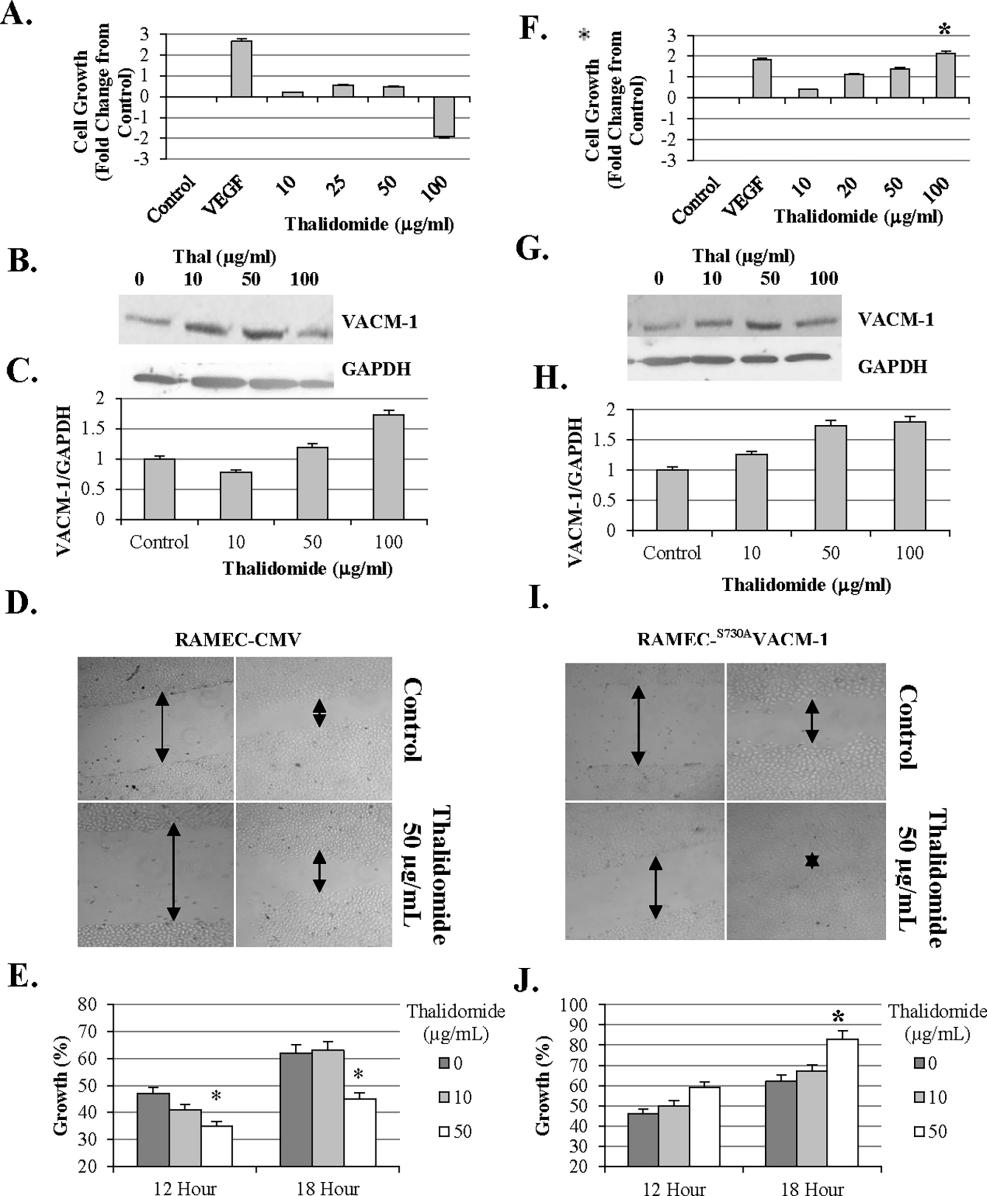
Thalidomide treatment inhibits growth in RAMEC but not in RAMEC transfected with a dominant negative mutant of CUL5 (^S730A^CUL5) cDNA. **A.** A representative growth assay results from CMV vector transfected RAMEC control cells treated with increasing doses of thalidomide. VEGF (50 nM) was used as a control. **B.** Western blot analysis of cell lysates from CMV vector transfected RAMEC treated with increasing doses of thalidomide for 24 hours. To ascertain equal protein loading blots were stripped and re-probed with anti-GAPDH specific antibody as described in *Methods*. **C.** The signal intensities shown in B above, were quantitated. **D.** A representative wound assay in CUL5 cDNA transfected cells treated with thalidomide. Arrows indicate space in the wound assay at time 0 and 18 hrs. **E.** Growth data shown in (D) was quantitated and expressed as a percent (%) regrowth from time 0. The effects of 0 μg/mL (black bars), 10 μg/ml (gray bars), and 50 μg/ml (white bars) of thalidomide were examined (*, p<0.05). **F.** A representative growth assay results from ^S730A^CUL5 cDNA transfected RAMEC cells treated with increasing doses of thalidomide. VEGF (50 nM) was used as a control. **G.** Dose dependent effect of thalidomide on CUL5 in ^S730A^CUL5 cDNA transfected RAMEC as detected with anti CUL5 protein specific antibody. **H**. Data shown in G were quantitated and expressed as mean ± standard error. (RAMEC CMV n = 2 and RAMEC−^S730A^VACM-1 (*n* = 3, * = *p* < 0.05). **I.** A representative light microscopy experiment using the wound assay in ^S730A^CUL5 cDNA transfected cells. Arrows indicate space in the wound assay. **J.** Growth data shown in (I) were quantitated and expressed as a percent regrowth from time 0. The effects of 0 (black bars), 10 μg/ml (gray bars), and 50 μg/ml (white bars) of thalidomide were examined (* = *p* < 0.05).

**Fig 8:**
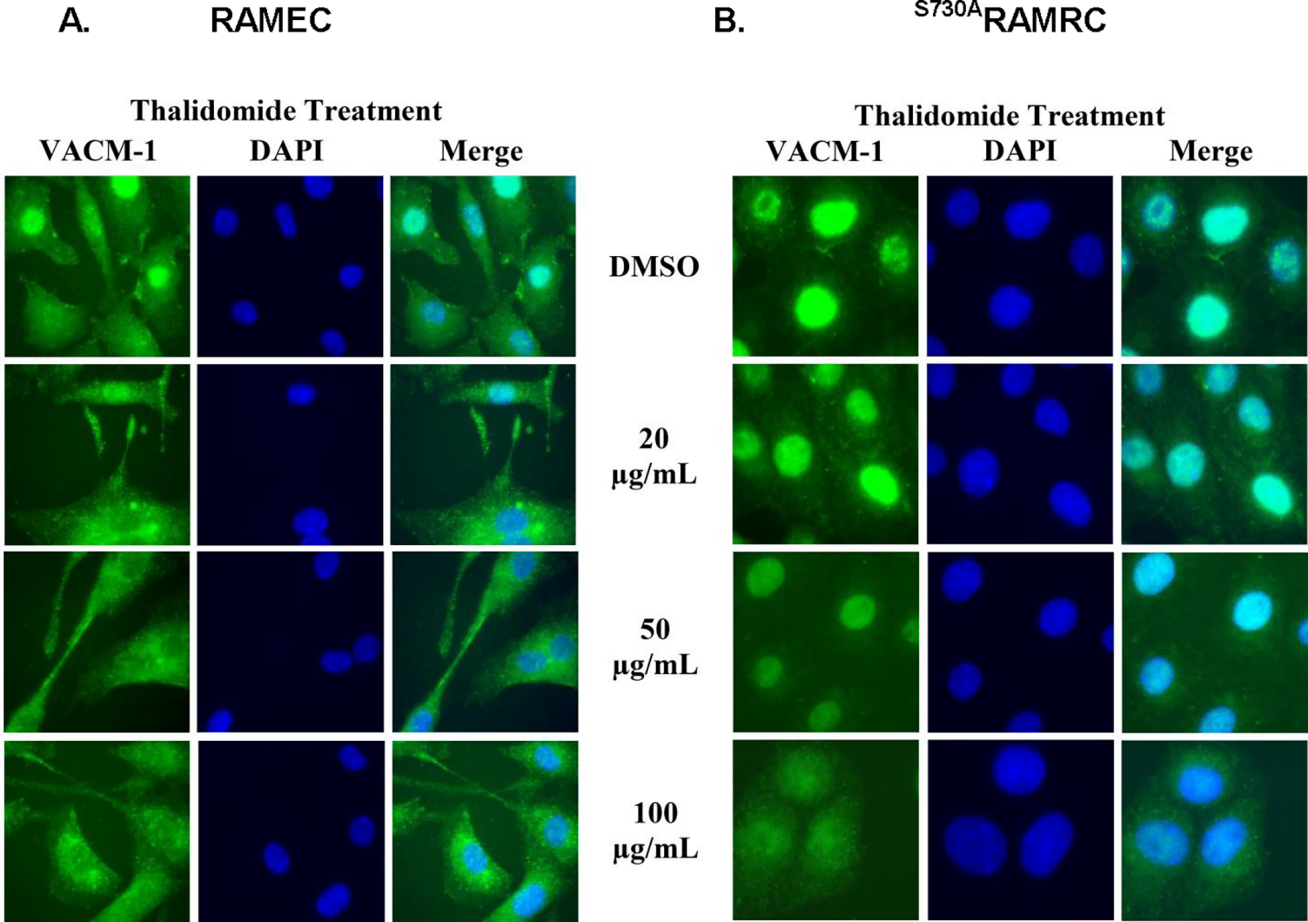
Effect of thalidomide on expression and cellular localization of CUL5 in control RAMEC and RAMEC transfected with ^S730A^CUL5. **A.** Immunocytochemistry of control RAMEC treated with thalidomide and immunostained with anti-CUL5 (VACM-1) Ab. **B.** ^S730A^CUL5 cDNA transfected RAMEC treated with thalidomide and immunostained with anti- CUL5 Ab. Cells were mounted in Vectashield^®^ containing DAPI (magnification, 100X).

Although rat tissue-derived endothelial cells may be less responsive to thalidomide [33, 35], inhibition of angiogenesis in rat aortic ring cultures and mouse corneal models has been reported [35] and thalidomide has been shown to compromise the development of rat skeletal muscle [41]. Further, the inhibition of endothelial cell proliferation by thalidomide may depend on the endothelial cell diversity [38]. In our previous work we have used rat endothelial cells (RAMEC) as a model to study CUL5-dependent signaling [23,45,46]. Specifically, our work demonstrated that a ^S730A^CUL5 mutant which cannot be phosphorylated by PKA, when expressed in RAMEC or COS-1 cells, is constitutively modified by NEDD8 and induces cellular proliferation [23]. Thus, we used this model to examine whether constitutive neddylation of CUL5 will affect thalidomide-dependent proliferation. First, the antiproliferative effect of thalidomide in RAMEC was established. As expected, higher doses of thalidomide were required to suppress cellular growth in control RAMEC when compared to the HUVEC (Fig 7A). To determine if these effects were associated with changes in CUL5 concentrations, we performed Western blot analysis of lysates collected from control and thalidomide treated RAMEC. When equal amount of protein was loaded on the gel, cells treated with 50 μg/mL thalidomide had higher CUL5 protein levels when compared to controls (Fig 7B and 7C). In addition to its effects on cell growth, the effect of thalidomide on cell migration was monitored using a wound assay. The results from these experiments showed that RAMEC treated with at least 50 μg/ml thalidomide grew slower when compared to the cells treated with the vehicle. Next, we examined the effect of thalidomide on RAMEC stably transfected with ^S730A^CUL5 [23,46]. Our data suggest that even at 100 μg/ml, thalidomide no longer inhibited growth (Fig 7F). Interestingly, cells treated with 50–100 μg/ml grew significantly faster when compared to their controls. Again, Western blot analysis of cell lysates from RAMEC transfected with ^S730A^CUL5 cDNA showed a small increase in VACM-1/CUL5 protein expression (Fig 7G and 7H). The wound assay results further confirmed the inability of thalidomide to inhibit growth of RAMEC transfected with the ^S730A^CUL5 cDNA. A significant increase in the wound growth in response to 50 μg/ml of thalidomide was observed 18 hours after treatment (Fig 7I and 7J). Cells were confluent 24 hours after treatment and therefore the differences in growth using the wound assay could not be quantitated. The specificity of this effect is supported by the observation that the proliferative effect of VEGF treatment was not compromised in ^S730A^CUL5 cDNA transfected cells (Fig 7F). To examine if thalidomide treatment affected concentration and/or localization of CUL5 protein in RAMEC, the immunocytochemistry experiments were performed in both cell lines. Our results showed that treatment with 50 μg/ml thalidomide decreased nuclear localization of CUL5 in the control cells but not in cells transfected with ^S730A^CUL5 cDNA where its localization remained nuclear (Fig 8A and 8B).

## Discussion

CUL5 was previously reported to regulate cellular proliferation and to act as a potential tumor suppressor [19–21]. Since a growing tumor requires nutrients acquired through an increase in blood flow and angiogenesis, regulation of vascular growth and permeability may have a direct impact on cancer [47–51]. The work presented here demonstrates that in human endothelial cells *in vitro*, the antiproliferative effect of thalidomide may depend on the presence of CUL5. Our results also show that thalidomide treatment is associated with abnormalities in cellular morphology and with redistribution of CUL5 from the nucleus to the cell cytosol (Figs 1 and 2). Whether this translocation of CUL5 may control previously described effects of thalidomide on actin polymerization, cytoskeletal rearrangement, lamellipodia formation, stress fiber formation and inhibition of cell migration [37] remains to be determined. Importantly, we show for the first time that the antiproliferative effect of thalidomide was significantly reduced or even reversed in cells transfected with anti-CUL5 siRNA (Fig 3). The involvement of CUL5 in the control of endothelial cell proliferation and its control by thalidomide is further underscored by the data demonstrating that anti-CUL5 siRNA transfection induced angiogenic phenotype when cells were grown on a Matrigel support (Fig 3E and 3F). Again, under those conditions, the inhibitory effect of thalidomide on cord-formation was reduced. Thus, in addition to CUL4A, required for the expression of tbx5a transcription factor essential during heart and limb development, and CUL2 required for vasculogenesis [51, 52], this work identifies CUL5 as another cullin that can regulate vasculogenic and angiogenic processes. We have previously demonstrated that overexpression of CUL5 in COS-1 cells decreases expression of AQP1 [25], thus acting indirectly to regulate water transport and vascular permeability. Together with these findings, this data supports a role for CUL5 in controlling vascular endothelial permeability.

The low expression or loss of CUL5 may also explain resistance in cancers not sensitive to thalidomide. Indeed, previous work has shown that silencing CUL5 expression reduced the sensitivity of several cancer cell types to three structurally distinct inhibitors of HSP90 [27]. This work might also provide a mechanism of action for thalidomide inhibition of HIV replication, since the control of CUL5 activity is essential for APOBEC3G degradation and the prevention of HIV infectivity [11–13] and CUL5 polymorphism has been associated with increased CD4^+^ T cell loss in HIV infected individuals [53]. Finally, thalidomide-induced inhibition of HIV type I replication [54] may also depend on CUL5.

All cullins are covalently modified at a conserved Lys residue at the carboxy-terminus by a ubiquitin-like protein, NEDD8 [6–9]. A continuous cycle of neddylation and deneddylation (Dcn1 and COP9/DEN1/CAND1 enzymes, respectively) may be essential for the activation of cullin-based ubiquitin ligases and is now an important target for anticancer drug development [55,56]. Further, the inhibition of neddylation by MLN4942, a specific inhibitor of NEDD8-activating enzyme (NAE), induces apoptosis, autophagy and decreases tumor angiogenesis in multiple melanoma [55,56]. Recently, a CUL4A-dependent ligase has been implicated in thalidomide-dependent anticancer effects [40–43] but the role of the neddylation in this process has not been explored. Since the neddylation process is now a target for developing novel compounds for the treatment of malignancies that include cancers, HIV infectivity and immunosuppression [54], identifying novel targets for these drugs is important. Our results suggest that in endothelial cells the antiproliferative effect of thalidomide may depend on its ability to control the neddylation status of CUL5 and/or its subsequent nuclear localization. Whether induction of NEDD8 by thalidomide targets unknown substrate(s) in the endothelium in the absence of CUL5 should be considered.

In conclusion, our results suggest that the antiproliferative effect of thalidomide in endothelial cells is dependent on the presence of CUL5 and that thalidomide may regulate neddylation of CUL5 and its localization to the nucleus. When CUL5 is decreased or constitutively neddylated, however, thalidomide is unable to inhibit cellular proliferation. Because endothelial cells are an essential barrier in all cancer types and viral infections, CUL5 may be an important new biomarker for predicting a specific response to thalidomide and possibly other anti-angiogenic drugs used clinically to treat disorders that include multiple myeloma and HIV infections.
